# Expression of Pentraxin 3 and Thrombospondin 1 in Gingival Crevicular Fluid during Wound Healing after Gingivectomy in Postorthodontic Patients

**DOI:** 10.1155/2016/4072543

**Published:** 2016-06-14

**Authors:** Anne Marie Rauten, Isabela Silosi, Stefan Ioan Stratul, Liliana Foia, Adrian Camen, Vasilica Toma, Daniel Cioloca, Valeriu Surlin, Petra Surlin, Maria Bogdan

**Affiliations:** ^1^Department of Orthodontics, University of Medicine and Pharmacy, Strada Petru Rares 2, 200349 Craiova, Romania; ^2^Department of Immunology, University of Medicine and Pharmacy, Strada Petru Rares 2, 200349 Craiova, Romania; ^3^Department of Periodontology, Victor Babes University of Medicine and Pharmacy, P-ta Eftimie Murgu 2A, 300041 Timisoara, Romania; ^4^Department of Biochemistry, Grigore T. Popa University of Medicine and Pharmacy, Strada Universitatii 16, 700115 Iasi, Romania; ^5^Department of Oral Surgery, University of Medicine and Pharmacy, Strada Petru Rares 2, 200349 Craiova, Romania; ^6^Department of Surgery, Grigore T. Popa University of Medicine and Pharmacy, Strada Universitatii 16, 700115 Iasi, Romania; ^7^Department of Surgery, University of Medicine and Pharmacy, Strada Petru Rares 2, 200349 Craiova, Romania; ^8^Department of Periodontology, University of Medicine and Pharmacy, Strada Petru Rares 2, 200349 Craiova, Romania; ^9^Department of Pharmacology, University of Medicine and Pharmacy, Strada Petru Rares 2, 200349 Craiova, Romania

## Abstract

*Background*. Wound healing is a tissue repair process after an injury, and two of its main components are inflammation and angiogenesis, in which course a cascade of mediators is involved. The aim of this research was to evaluate the involvement of Pentraxin 3 and Thrombospondin 1 in wound healing after periodontal surgery (gingivectomy) for gingival overgrowth during orthodontic treatment with or without magnification devices, by assessing their levels in GCF.* Methods*. From 19 patients with gingival overgrowth as a result of fixed orthodontic treatment, the overgrown gingiva was removed by gingivectomy, from one half of the mandibular arch without magnification and from the other under magnification. Pentraxin 3 and Thrombospondin 1 were determined from gingival crevicular fluid by ELISA tests.* Results*. Statistically significant differences (*p* < 0.05) and correlations between levels of the two biomarkers were analyzed. Statistically significant differences were established between levels of the two biomarkers at different time points, with significant positive correlation at the point of 24 hours.* Conclusions*. Within the limitations of this study, the results seem to sustain the involvement of Pentraxin 3 and Thrombospondin 1 in the processes of inflammation and angiogenesis in wound healing of patients with postorthodontic gingivectomy. The dynamics of Pentraxin 3 and Thrombospondin 1 levels could suggest a reduced inflammation and a faster angiogenesis using microsurgery.

## 1. Introduction

As an exudate that reflects with fidelity the events in the periodontium, the gingival crevicular fluid (GCF) may be used to determine the levels of certain biomarkers [1]. Expressions of biologically active substances in GCF in periodontal disease and its nonsurgical treatment [2–4], during initial phase of orthodontic tooth movement [5–7], or after various periodontal surgical techniques [8] were highlighted so far.

Today, gingivectomy remains the oldest and one of the basic periodontal surgical procedures [9], without vertical incisions and sutures which are considered to determine local inflammation [10, 11]. The development of more sophisticated flap methods have relegated the gingivectomy to a lesser role in the current repertoire of available techniques; between its limited indications remained the resection of gingival enlargement, including gingival overgrowth (GO) as a result of orthodontic treatment. Most of the time, the removal of GO is performed at the end of the orthodontic treatment, when GO does not regress spontaneously [12].

Although periodontal microsurgery presumes previous training of the operator, skills, expensive magnification devices, specific surgical instruments, and longer operative time, it is often used in practice due to its clinical advantages determined by the good postoperative clinical course: a faster healing of the gingival mucosa, reduced signs of local inflammation, and less pain for the patient [13]. These clinical advantages of periodontal microsurgery were studied during the coverage of gingival recessions [14] and the regenerative surgical treatment of infraosseous defects [15].

Similar results were found for other tissues, in which healing was faster after microsurgery [16]. There are studies trying to bring scientific proofs for the clinical observations by determining the levels of matrix metalloproteinase 2 (MMP2), matrix metalloproteinase 9 (MMP9), TGF-1beta, and TNF alpha [17, 18] to emphasize the role of microsurgical approach in reducing the inflammation and stimulating the angiogenesis involved in the healing process.

Wound healing is a tissue repair process after an injury, and two of its main components are inflammation and angiogenesis, in which course a cascade of mediators is involved [19].

Pentraxin 3 (PTX3) is an acute phase protein that belongs to the pentraxin superfamily (with C reactive protein, CRP, and serum protein A, SAP) and is considered to be a marker of inflammation [20, 21]. PTX3 is a long pentraxin produced especially by fibroblasts and neutrophils [22, 23]. The levels of PTX3 in GCF in periodontal health and disease or during orthodontic treatment were estimated in previous studies [24, 25]. On the other hand, there are findings that link the PTX3 expression to fibroblast growth factor 2 (FGF2) suggesting the involvement of PTX3 in angiogenesis downregulating [26, 27].

Thrombospondin 1 (TSP1) is a glycoprotein and is one of the endogenous inhibitors of angiogenesis, of which main antiangiogenic site is type I collagen repeats, active both as whole molecule and as fragments [28]. Implication of TSP1 in inhibiting the angiogenesis is by direct mechanism through interaction with specific receptors and indirect by influencing the activity of other mediators of angiogenesis [29].

The aim of this research was to (1) evaluate the involvement of PTX3 and TSP1 in wound healing after gingivectomy with or without microsurgical instruments and magnification devices, by assessing their levels in GCF and (2) evaluate if the more favorable clinical course of wound healing in case of microsurgery reported by other previous studies could be explained through the different dynamics of PTX3 and TSP1 levels.

## 2. Materials and Methods

### 2.1. Subjects

19 patients aged between 14 and 32 years (mean age 18.42 ± 5.46) were included (11 women and 8 men). All patients presented with gingival overgrowth with maximum score of 2 [30] as a result of fixed orthodontic treatment of the mandibular arch, after 1 month of contention. The ethical approval was given by the Committee of Ethics, Academic and Scientific Deontology of University of Medicine and Pharmacy, Craiova, Romania, and patients gave informed consent. Inclusion criteria were as follows: (1) gingival overgrowth including at least 6 teeth in the mandibular anterior zone; (2) no attachment loss; (3) no radiographic sign of bone loss. Exclusion criteria were as follows: (1) systemic diseases and treatments leading to gingival hypertrophy; (2) anti-inflammatory treatments during the last 30 days; (3) antibiotic treatment in the last 3 months; (4) pregnancy. All patients were nonsmokers. One week before surgery, all patients underwent a session of ultrasonic scaling and received indications regarding the oral hygiene to obtain a good control of the bacterial plaque: dental brushing twice a day with the same toothpaste available on the market (Colgate Total, USA) and mouth rinsing with chlorhexidine CHX 0.12% twice a day, 30 min after teeth brushing.

### 2.2. Surgery

The overgrown gingiva was removed from one half of the mandibular arch by gingivectomy [9], without microsurgical instruments and without magnification, and from the other half by the same surgical technique performed under magnification by using a dental microscope (Seliga, Seliga Microscopes Sp. z.o.o., Lodz, Poland), microsurgical blades, and microsurgical instruments (Hu Friedy, Chicago, IL, USA, by RED Intl). Prior to the surgery, the depths of the false pockets were identified by probing with a periodontal probe (UNC15, Hu Friedy, Chicago, IL, USA, by RED Intl). External bevel scalloped incisions one millimetre coronally from the base of the false pockets were performed. Haemostasis was achieved by gentle gauze packing. Surgeries were performed in the morning, between 10.00 and 11.00 a.m., 2 hours after domestic hygiene and 1 hour after first sampling of GCF. After the procedure, patients were recommended to maintain hydration in the first 24 h with nonacidulated sugar-free beverages and to stay on a liquid diet. The same indications for oral hygiene were maintained for the next 2 weeks, except the 2nd brushing on the day of surgery.

### 2.3. GCF Sampling

Samples of GCF were obtained (one sample/site/tooth) using paper strips (PerioPaper, Oraflow Inc., Smithtown, NY, USA), maintained for 30 sec in the gingival sulcus, at 1 minute interval [31], from a mandibular tooth with gingivectomy performed with microsurgical instruments under magnification, test tooth M (TTM), and without microsurgical instruments and no magnification, test tooth G (TTG), and from a maxillary tooth without hypertrophy and no gingivectomy, control tooth (CT). Prior to the procedure, the supragingival plaque was gently removed with a Gracey curette (HU Friedy, IL, USA) and the area was isolated with cotton rolls while using a saliva ejector. Samplings were done 1 hour before gingivectomy (considered as baseline level of the two markers) and at 24, 72, and 120 hours and 1 and 2 weeks after gingivectomy, in the morning, and 1 hour after the first mouth hygiene of the day. The method of GCF collection and the calculation of the concentration were performed as previously described [32]. In short, the harvested GCF volume was measured with a precalibrated device (Periotron 8000, Oraflow Inc., Smithtown, NY, USA) designed to measure volumes of 10^−6^ L (*μ*L) and samples were introduced in polypropylene tubes with 100 *μ*L PBS and stored at −20°C prior to their use. In the case of saliva or blood contamination, the sample was discarded and repeated in another site with gingivectomy. Calculation of the concentration in each sample was performed by dividing the amount of substances by the volume of the sample (ng/mL).

### 2.4. Immunological Assays in GCF

For quantitative determination in GCF, commercial kits Quantikine Elisa Kit (R&D Systems, USA) for PTX3 (test sensitivity 0.116 ng/mL) and TSP1 (test sensitivity 0.944 ng/mL) were used. Every component of the kit was used according to the manufacturer's indications. Reading was performed at 450 nm with a correction at 540 nm to reduce optical imperfections on the reading plate.

### 2.5. Statistical Analysis

Statistical analysis was performed with a dedicated software (SPSS 16.0, Chicago, IL, USA). The mean ± standard deviation (M ± SD) was used as primary data. The results were statistically analyzed using the nonparametric Mann-Whitney *U* test (*p* < 0.05 for statistical significance). Bonferroni correction was applied. Pearson correlation coefficient was used to verify if there was correlation between levels of PTX3 and TSP1.

## 3. Results

**Figure 1:**
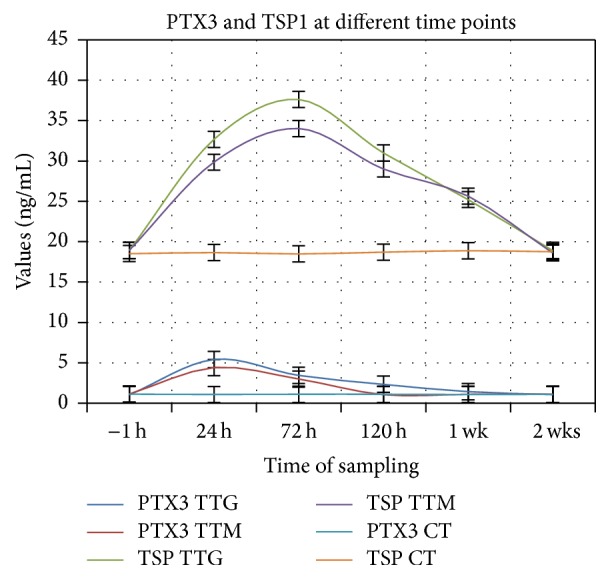
Dynamics in time of PTX3 and TSP1 levels in TTG and TTM. Levels in ng/mL, M ± SD. Maximum levels for PTX3 at 24 h and for TSP1 at 72 h in both TTM and TTG.

The results obtained for the TTG showed an increase of GCF levels of PTX3 from 1.05 ng/mL 1 hour before gingivectomy (−1 h as baseline level) to a maximum of 5.43 ng/mL (approximately 5-fold compared to the baseline level) at 24 hours, followed by a decrease reaching 1.08 ng/mL at 2 weeks (Figure 1).

In the TTM group, the results showed an increase of PTX3 values from 1.12 ng/mL at 1 h before gingivectomy (−1 h at baseline level) to a maximum of 4.42 ng/mL (approximately 4-fold compared to the baseline level) at 24 hours, followed by a decrease reaching the baseline value 1.05 ng/mL at 120 hours, and were maintained at this level after 1 and 2 weeks (Figure 1).

For the TSP1 levels in TTG, an increase of values from 18.91 ng/mL 1 hour before gingivectomy (−1 h as baseline level) to a maximum of 37.84 ng/mL (approximately 2-fold compared to the baseline level) at 72 hours and a decrease to 18.91 ng/mL at 2 weeks (Figure 1) were noted.

For the TSP1 levels in TTM, an increase from 18.93 ng/mL 1 hour before gingivectomy (−1 h as baseline level) to a maximum of 34 ng/mL (approximately 1.7-fold compared to the baseline level) at 72 hours and a decrease to 18.61 ng/mL at 2 weeks (Figure 1) were noted.

**Figure 2:**
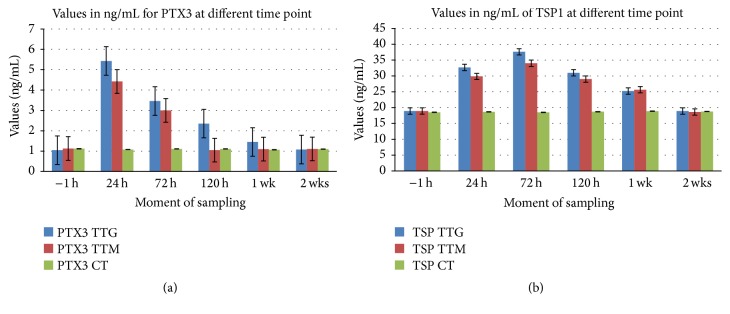
Differences between levels of PTX3 and TSP1 in TTG and TTM. (a) Statistically significant differences between PTX3 at 24, 72, and 120 h and baseline in TTG and statistically significant differences between PTX3 at 24 and 72 h and baseline in TTM; (b) statistically significant differences between TSP1 at 24, 72, and 120 h and 1 wk and baseline in TTG and statistically significant differences between TSP1 at 24, 72, and 120 h and 1 wk and baseline in TTM.

Statistically significant differences (*p* < 0.05) were found between mean levels of PTX3 in TTG at 24, 72, and 120 hours and the baseline level. There was no statistically significant difference (*p* > 0.05) between the levels at 1 week and 2 weeks and the baseline level. Statistically significant differences (*p* < 0.05) were found between mean levels of PTX3 in TTM at 24 and 72 hours and the baseline level. There were no statistically significant differences (*p* > 0.05) between the levels at 120 hours, 1 week, and 2 weeks and the baseline level. Levels of PTX3 in TTG were higher as in TTM at 24, 72, and 120 hours and 1 week, statistically significant differences (*p* < 0.05) being found between mean levels of PTX3 in TTG and TTM at 24 (maximum levels for both TTG and TTM) and 120 hours (levels in TTM reaching the baseline level). There was no statistically significant difference (*p* > 0.05) between the levels of PTX3 in TTG and TTM at baseline (−1 h), 72 hours, 1 week, and 2 weeks (Figure 2(a)).

Statistically significant differences (*p* < 0.05) were found between mean levels of TSP1 in TTG at 24, 72, and 120 hours and 1 week and the baseline level. There was no statistically significant difference (*p* > 0.05) between the levels at 2 weeks and the baseline level. Statistically significant differences (*p* < 0.05) were found between mean levels of TSP1 in TTM at 24, 72, and 120 hours and 1 week and the baseline level. There was no statistically significant difference (*p* > 0.05) between the levels at 2 weeks and the baseline level. Levels of TSP1 in TTG were higher as in TTM at 24, 72, and 120 hours, statistically significant differences (*p* < 0.05) being found between mean levels of TSP1 in TTG and TTM at 24, 72 (maximum levels for both TTG and TTM), and 120 hours. There was no statistically significant difference (*p* > 0.05) between the levels of TSP1 in TTG and TTM at baseline (−1 h), 1 week, and 2 weeks. (Figure 2(b)).

For both test groups, TTG and TTM, there were significant positive correlations between levels of PTX3 and TSP1 at 24 hours and poor correlations between levels of PTX3 and TSP1 at 72 and 120 hours, 1 week, and 2 weeks (Tables 1 and 2).

## 4. Discussion

Wound healing is a complex biological process that consists of four phases: haemostasis, inflammation, proliferation, and remodelling of tissues [33]. Immediately after injury, the haemostasis begins, and proinflammatory cytokines and growth factors like TGF-beta, epithelial growth factor (EGF), VEGF, and fibroblasts growth factor (FGF) [34, 35] are released; thus the inflammatory stage is promoted. During this stage, macrophages and neutrophils infiltrate the wound bed and release other promoter cells of inflammation [36]. Angiogenesis is a part of the proliferation phase and is characterized by the formation of new blood vessels [37]. The final phase is the tissular remodelling; the vascular density in the wound returns to normal; this process could continue for years [35]. Angiogenesis and inflammation are two important processes for wound healing; investigation of their specific markers may offer information about its progression [19].

A recent narrative review on the fundamental clinical principles in periodontal plastic surgery and mucosal augmentation concludes that delicate tissue handling and tension-free wound closure represent prerequisites for optimal healing outcomes. Such procedures benefit nowadays from microsurgical techniques and magnification devices [38].

The clinical advantages of microsurgical instruments, techniques, and work under magnification in periodontology were first mentioned in the founding studies of Tibbetts and Shanelec [39] and Burkhardt and Hürzeler [40]. The same microsurgical approach was further designated as MIST (Minimally Invasive Surgical Technique) and used enamel matrix derivative as a regeneration agent in intrabony defects, with the observation of the same clinical excellent results [41–43].

Wound healing characteristics in periodontal surgery for different surgical techniques were assessed especially by clinical parameters. Comparing the periodontal micro- and macrosurgery, clinical criteria were used, which indicated for microsurgery an increased comfort and gain in attachment in case of regenerative treatment of infraosseous lesions treatment [15, 44] or reduction of gingival recessions by plastic microsurgery [44–48]. Gingivectomy is the simplest technique of periodontal surgery, offering a good access and high predictability [9]; it is performed without vertical incisions or sutures, which are known to promote local inflammation [10, 11].

In the present study, the gingival enlargement as a result of orthodontic treatment, without attachment or bone loss, was excised by gingivectomy performed 1 month after cessation of orthodontic forces, to avoid the influence of the orthodontic tooth movements on the secretion of biomarkers in GCF, and 1 week after scaling, under continuous motivation for oral hygiene and a good control of plaque. Expression of certain biomarkers of inflammation and angiogenesis in GCF was used to assess the characteristics of the wound healing after using microsurgical instruments and the dental operating microscope in performing this simple technique.

The involvement of PTX3 in some general diseases characterized by an inflammatory status was proven in several studies. Elevated plasma levels of PTX3 were found in the dengue virus infection [20] and also in the severe meningococcal disease [49]. PTX3 association with sepsis was proven, and the effect of antioxidant treatment on PTX3 levels was demonstrated [21]. The implication of PTX3 in the innate immunity [50] was studied, and levels of PTX3 are considered a marker of inflammation in psoriasis, as well [51]. The involvement of PTX3 in angiogenesis was also demonstrated. A FGF2-binding domain in the N-terminal extension of PTX3 spanning the PTX3-[97–110] region was identified, pointing to a novel function for the N-terminal extension of PTX3 and underlining the complexity of the PTX3 molecule for modular humoral pattern recognition [27]. This suggests that PTX3 may exert a modulatory function by limiting the angiogenic activity of FGF2 [52], FGF2 being found in human and porcine wound fluid, particularly at early stages after injuries [34, 53–55].

Thrombospondin 1 (TSP1) is a glycoprotein and is one of the endogenous inhibitors of angiogenesis with multiple active domains [29]. The ability of TSP1 to inhibit vascular growth appears to be located within the last 2 type 1 repeats. Therefore, inhibition of binding or further sequestration of FGF2 is the most likely mechanism of action of this amino-terminal portion of the type 1 repeats [28, 56]. These findings may support the idea that between the levels of PTX3 and TSP1 may be a correlation, both biomarkers being involved in limiting the angiogenic action of FGF2.

In the present study, there were no significant differences between the levels of PTX3 and TSP1 levels for CT and TTM or TTG before surgery (−1 h as baseline). Even if, for TTM and TTG, HTG was present, scaling was performed 1 week before surgery, and oral hygiene was good, the HTG did not display any clinical signs of inflammation, explaining the lack of significant differences between CT, TTM, and TTG. PTX3 levels increase in the first 24 hours, starting to decrease afterwards for both types of surgery, but the levels are lower in case of microsurgery compared to gingivectomy without magnification, indicating a lesser intense inflammatory reaction.

Reduction of PTX3 levels after 24 h is faster and more abrupt in case of microsurgery, reaching levels before intervention at 120 h, while, for gingivectomy without magnification, levels before surgery are reached at 1 week. These findings could suggest that inflammation of shorter intensity and duration in case of microsurgery may explain the higher comfort of the patient reported by some authors [13]. Reduced inflammatory reaction could be explained by less mechanical trauma given by the use of a microsurgical approach, including microsurgical instruments, and so an improvement of wound healing is obtained [38].

TSP1 reaches maximal values at 72 hours from intervention in both situations after a continuous increase, but as for PTX3, levels in case of microsurgery are lower than that of gingivectomy without magnification. In case of microsurgery, TSP1 level reaches the value before intervention faster than in case of gingivectomy without magnification, which may suggest that angiogenesis is faster in this case and so is the wound healing.

The results of this study show a stronger correlation between the PTX3 and TSP1 levels at 24 h from surgical intervention for both types of surgery, possibly related to the expression of FGF2, which is reported to be maximal in the first hours from injury [31]. Future studies determining pro- and antiangiogenic markers, including different FGFs, could bring new insights to this possible correlation.

Further studies assessing the expression of different biomarkers possibly involved in wound healing after more sophisticated usual surgical techniques could correlate the results of the present study to other factors and find the place occupied by these substances in the mediators' fall that is activated during this complex process.

## 5. Conclusions

Within the limitations of this study, the results seem to sustain the involvement of PTX3 and TSP1 in the processes of inflammation and angiogenesis in wound healing in patients with postorthodontic gingivectomy, showing a change in time of PTX3 and TSP1 levels in GCF of these patients, regardless of the use of microsurgical instruments and magnification of a dental microscope. The dynamics of PTX3 and TSP1 levels suggest a reduced inflammation and a faster angiogenesis using microsurgery, which could explain the more favorable clinical course of wound healing reported by other previous studies.

## Figures and Tables

**Table 1:** Correlations between PTX3 and TSP1 levels at different time points in TTM. For the test group TTM there were significant positive correlations between levels of PTX3 and TSP1 at 24 hours (*r* = 0.972, *p* < 0.05) and poor correlations between levels of PTX3 and TSP1 at 72 and 120 hours, 1 week, and 2 weeks (Pearson test for correlations).

TSP1 levels in TTM	PTX3 levels in TTM					
	−1 h	24 h	72 h	120 h	1 wk	2 wks
−1 h	*r* = 0.113	—	—	—	—	—
24 h	—	**r** = 0.972	—	—	—	—
72 h	—	—	*r* = 0.474	—	—	—
120 h	—	—	—	*r* = 0.431	—	—
1 wk	—	—	—	—	*r* = 0.37	—
2 wks	—	—	—	—	—	*r* = 0.331

**Table 2:** Correlations between PTX3 and TSP1 levels at different time points in TTG. For the test groups TTG there were significant positive correlations between levels of PTX3 and TSP1 at 24 hours (*r* = 0.984, *p* < 0.05 for TTG) and poor correlations between levels of PTX3 and TSP1 at 72 and 120 hours, 1 week, and 2 weeks (Pearson test for correlations).

TSP1 levels in TTG	PTX3 levels in TTG					
	−1 h	24 h	72 h	120 h	1 wk	2 wks
−1 h	*r* = 0.032	—	—	—	—	—
24 h	—	**r **=** 0.984**	—	—	—	—
72 h	—	—	*r* = 0.42	—	—	—
120 h	—	—	—	*r* = 0.593	—	—
1 wk	—	—	—	—	*r* = 0.564	—
2 wks	—	—	—	—	—	*r* = 0.002
